# Facilitating arrhythmia simulation: the method of quantitative cellular automata modeling and parallel running

**DOI:** 10.1186/1475-925X-3-29

**Published:** 2004-08-30

**Authors:** Hao Zhu, Yan Sun, Gunaretnam Rajagopal, Adrian Mondry, Pawan Dhar

**Affiliations:** 1 Systems Biology Group, Bioinformatics Institute, Biopolis Street, 138671, Singapore; 2 Medical Informatics Group, Bioinformatics Institute, Biopolis Street, 138671, Singapore

## Abstract

**Background:**

Many arrhythmias are triggered by abnormal electrical activity at the ionic channel and cell level, and then evolve spatio-temporally within the heart. To understand arrhythmias better and to diagnose them more precisely by their ECG waveforms, a whole-heart model is required to explore the association between the massively parallel activities at the channel/cell level and the integrative electrophysiological phenomena at organ level.

**Methods:**

We have developed a method to build large-scale electrophysiological models by using extended cellular automata, and to run such models on a cluster of shared memory machines. We describe here the method, including the extension of a language-based cellular automaton to implement quantitative computing, the building of a whole-heart model with Visible Human Project data, the parallelization of the model on a cluster of shared memory computers with OpenMP and MPI hybrid programming, and a simulation algorithm that links cellular activity with the ECG.

**Results:**

We demonstrate that electrical activities at channel, cell, and organ levels can be traced and captured conveniently in our extended cellular automaton system. Examples of some ECG waveforms simulated with a 2-D slice are given to support the ECG simulation algorithm. A performance evaluation of the 3-D model on a four-node cluster is also given.

**Conclusions:**

Quantitative multicellular modeling with extended cellular automata is a highly efficient and widely applicable method to weave experimental data at different levels into computational models. This process can be used to investigate complex and collective biological activities that can be described neither by their governing differentiation equations nor by discrete parallel computation. Transparent cluster computing is a convenient and effective method to make time-consuming simulation feasible. Arrhythmias, as a typical case, can be effectively simulated with the methods described.

## Background

Arrhythmias, a significant direct cause of death in heart diseases, are emergent and evolvable events that come with little prior warnings and allow limited response time [1]. Although ECG waveforms – the mapping of body surface potentials of cardiac cells – have routinely been used to diagnose arrhythmias, as integrated signals they tell us little about what happen at cell and ionic channel levels. They therefore are only marginally useful in guiding the clinical use of anti-arrhythmic drugs to treat disturbed cardioelectrical activity at cell and channel levels. Many of such drugs used today are ionic channel blocking agents [2].

To overcome the inherent limitations of clinical investigation, computational modeling and simulation has been widely recognized as a valuable alternative approach. Traditionally, cardiac modeling has centered on ECG simulation. Using the finite element method (FEM), the virtual heart and whole chest are partitioned into numerous elements representing a group of cells. The ECG is then simulated, based on computing the body surface potential of each cardiac element [3-5]. Basically, this method does not concentrate on cellular electrophysiological issues at the channel level, and thus fails to precisely associate macro level phenomena (ECG waveforms) with micro level activities and to make use of the considerable knowledge of cellular electrophysiology accumulated over the past decades. To improve the understanding of arrhythmias and to find effective perturbations, electrophysiological modeling using membrane equations is required so that mechanisms of arrhythmias at cell, channel, and even molecular levels can be investigated [6-8].

To study arrhythmias using a large-scale realistic electrophysiological model, two issues need to be effectively resolved: model building and operation. Though the widespread paradigm of modeling with C or C++ remains a workable choice, the huge number of cardiac cells in a realistic three-dimensional (3-D) whole-heart model and the numerous modifications of the model to simulate various pathological conditions, make more desirable efficient modeling based on transparent parallel computing. To build and run a model with parallel computing technologies, two strategies were separately developed in recent years. To provide transparent and parallel descriptions, cellular automata were used [9-11,50]; for efficient execution, distributed computing was adopted [12,13]. Yet, each strategy alone is not sufficient for the successful simulation of arrhythmias. On the one hand cardiac models built with traditional cellular automata are qualitative, and thus do not use the Hodgkin-Huxley (HH) action potential equations to describe channel electrical activity. Consequently, many arrhythmias, such as those triggered by early-after-depolarization (EAD) and delayed-after-depolarization (DAD), can not be simulated. On the other hand, physical parallelism on parallel computers is also not fully exploited in these cellular automata models. MPI, the programming protocol for distributed-memory multiprocessors (DMP), and OpenMP, the programming protocol for shared-memory multiprocessors (SMP), are not used [14,15]. Partly because of these two issues, arrhythmia simulations with a realistic whole-heart electrophysiological model have not been fruitfully conducted.

There is compelling evidence that cardiomyocytes are not arranged in a uniformly connected continuum, as has often been assumed and simulated in the past. Along with nonlinear ionic channel electrical activity, discontinuous electrical propagation is another key feature of cardioelectrical activity [16]. The former demands a precise membrane equation based description; the latter requires a gap junction based discrete model. To efficiently build heterogeneous models containing different types of cardiomyocytes described by different membrane equation models and connected through different gap junctions remains a major challenge. In the present study, we propose a method using an extended, quantitative cellular automaton to build a discrete whole heart model with the data of the Visible Human Project (VHP) male cadaver [17]. An ECG simulation algorithm based on the membrane potential of each and every cell is designed and validated in a 2-D model built with the same method. Moreover, we combine cellular automata modeling with distributed parallel computing to realize efficient and affordable simulation. The parallel numerical solutions of the HH equations within a large number of cardiac cells are executed in parallel on a cluster with hybrid MPI and OpenMP programming. The parallel programming does not have to be manually coded in models built with the extended cellular automaton, because the modified compiler of the cellular automaton can automatically parallelize the codes, making parallelism fully transparent. The aim of this paper is to introduce the method and the whole-heart electrophysiological model. Results of performance evaluation on a four-node cluster are given. Based on this work, we conclude that quantitative modeling using extended and cluster computing enabled cellular automata is feasible and efficient, seamlessly binding conceptual and physical parallel computation. This method is suitable for a variety of computational intensive, tissue level modeling and simulation.

## Methods

### Cellular automata style quantitative computing

Cellular automata were first introduced by John von Neumann and Stanislaw Ulam in the 1940s, and gradually used to solve a wide range of problems, including multicellular biological modeling in which a natural correspondence between each automaton cell and each biological cell is assumed [18-21]. Simple local interaction producing complex global behavior is common to a variety of natural phenomena, including cardioelectrical activity. Though traditionally cellular automata are regarded as discrete parallel systems, new functions can be obtained with non-standard implementations [22,23]. For language-based cellular automata, a program encoded in a language instead of a rule is shared by all cells and describes the behavior of each cell. A compiler translates the cell program into executable files. To enhance the portability of the language, a two-step compilation is usually adopted, with C/C++ files being the intermediate codes, allowing the extension of such cellular automata systems.

**Figure 1:**
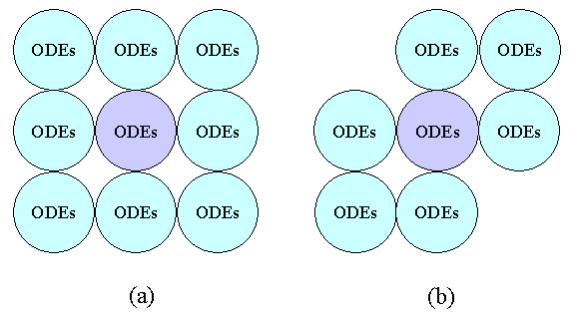
**Quantitative cellular automata with different neighborhoods. **(a) Moor neighborhood. (b) A user-defined neighborhood. The radius in both cases is 1.

*Cellular *is a cellular automata system based on the language *Cellang *[23], whose cell program comprises three parts: constant declarations, a cell declaration, and statements. The cell declaration defines a set of fields to store states of each cell between successive steps of computation. The cell array is described in a separate data file, whose output is piped to the cell program to provide the value of fields in each cell so as to enable computation. A predefined unchangeable variable *time* synchronizes the running of all cells. By conventions, in cellular automata computation quantitative computing is not fully supported and function call is not allowed. To overcome this inherent inadequacy of *Cellang *in quantitative computing and enable it to solve HH type membrane equations, we have added new language facilities, including the floating-point data type and the mathematical functions provided in the C libraries. Thus, *Cellang *is able to encode numerical solutions of the HH equations (Figure 1). A built-in function position() is also added to specify the global coordinates of the currently running cell. This function, in combination with the if-then statement, allows position-and time-dependent runtime perturbations to any cells, a function valuable for arrhythmia simulation. Viewing facilities are also extended to monitor and display simulation of electrical activitys at channel, cell, and organ level.

Running a model may be no easier than building it. The large number of cells, the small time step Δt and the long running time for arrhythmia simulation make it impractical to run a whole heart model on any low-end computer. Since large-scale SMP machines are extremely expensive, the prevailing parallel computing platforms are clusters of small-scale SMP or DMP, and the protocols of programming in Fortran, C, and C++ on such computers are OpenMP and MPI. First, an endless *while *loop is used to realize iterative computation. Second, since for an *n*-dimensional model each field in the cell program is translated into an *n*-dimensional data array in the intermediate C program, within the *while *loop *n *successive *for *loops are employed to traverse the *n *dimensional cell space. Only the codes within the *for *loops, which are statements in the cell program, need to be parallelized. Instead of defining one data array with an offset for each field in the original *Cellular *system, we use two data arrays in a flip-flop manner to support parallel write operation. OpenMP provides a group of directives to be inserted into the C program to tell the compiler the region to be executed in parallel. Such directives appear before the *for *loops to dispatch the outermost *for *loop into a group of threads, whose number is set dynamically according to the available CPUs. The simulation of cellular activity is thus implicitly parallelized in a shared memory space. To parallelize a *Cellang *program on a platform with distributed memory, explicit cell space decomposition is inevitable. Every data array is equally divided into several subsets located in distributed memory bodies, and computed by autonomous computing nodes that communicate each other through MPI. Since a cell needs to access its neighboring cells over the maximal radius *m *to compute transjunctional currents, each subset should enclose *m *extra layers of cells on each side to ensure that cells at the boundary layer(s) can access correct neighbors that are located in the outermost *m *layers of the two neighboring subsets, respectively. Field values of cells in these extra layers should be swapped between neighboring subsets after each round of computation to ensure that cells access updated data. Such data exchange is the major factor for the excessive time needed by cellular automata models running on distributed memory platforms. If cells have many fields, the time spent on such operations may even offset the benefits of parallelism.

**Figure 2:**
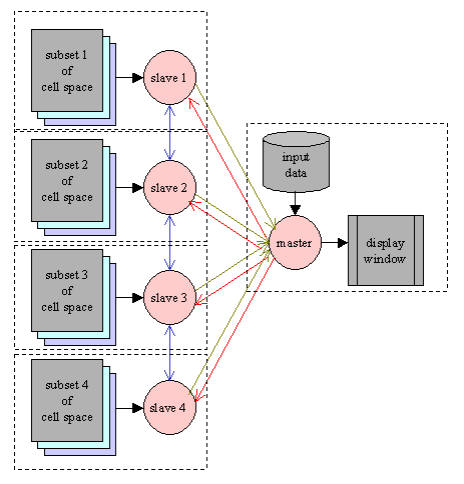
The master-slave structure of distributed computing with MPI programming.

To reach the maximal flexibility and portability for MPI-parallelization, a *master-slave *program structure is adopted (Figure 2). The number of *slaves *is set in the compilation command with an argument. The *master *is responsible for:

• Reading data from the data file into the cell space and dispatching the cell space at time step 0;

• collecting the value of a selected field in each cell from all *slaves *and displaying them at each time step;

• updating the variable *time *and sending its new value to every *slave *to trigger the next round of computation;

• doing some global, non-cellular automata style computing such as ECG simulation;

Each *slave *process is responsible for:

• receiving a subset of the cell space at time 0;

• exchanging the boundary layers with neighboring *slaves *at each time step;

• executing the cell program of cells in its subset at each time step;

• sending the value of a selected field to the *master *at each time step for display;

• receiving the new value of *time *at each time step.

Finally, to run the model on a cluster of SMP nodes, a two-tier parallelism – a coarse-grained setting among nodes and a fine-grained setting within nodes – is applied. The hybrid programming is straightforward – insert OpenMP directives into the *master *and *slaves*, as in the case of pure OpenMP programming to spawn a group of threads within each node to implement cell level parallelism. Due to the stereotyped appearance of these codes, we let the compiler generate them each time we compile a model, leaving the number of *slaves *and threads within each *slave *as command line arguments, so as to make parallel computation entirely transparent to users.

### Construction of the anatomical model of the heart

The anatomical model, which is a data file describing the distribution of the cell array and the initial value of fields in each cell, is independent of the cell program. The data file can be manually edited or generated by a program coded in C/C++. Due to the correspondence between each automaton cell and each biological cell, the building of the anatomical model is straightforward, even if a model has an irregular structure and a heterogeneous cell population. Usually, the Moore neighborhood is adopted, as is the case in our model.

**Figure 3:**
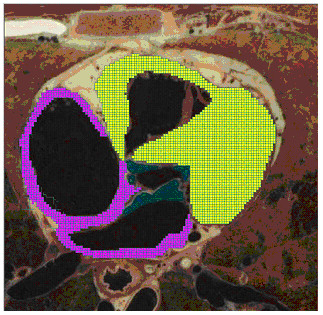
**Digitalizing slices of the digital male cadaver. **Yellow color indicates ventricular tissue, and pink color indicates atrial tissue.

**Figure 6:**
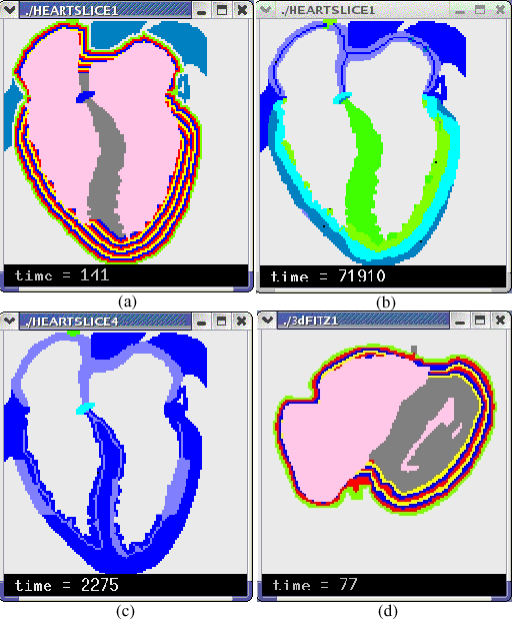
**The stratified heart walls. **(a) The stratified 2-D model. (b) The epicardium-to-endocardium repolarization in the 2-D model based on stratified cardiac walls. Different color indicates different transmembrane potential. (c) Two ischemia areas based on stratified cardiac walls. (d) The stratified 3-D model (a section).

**Figure 7:**
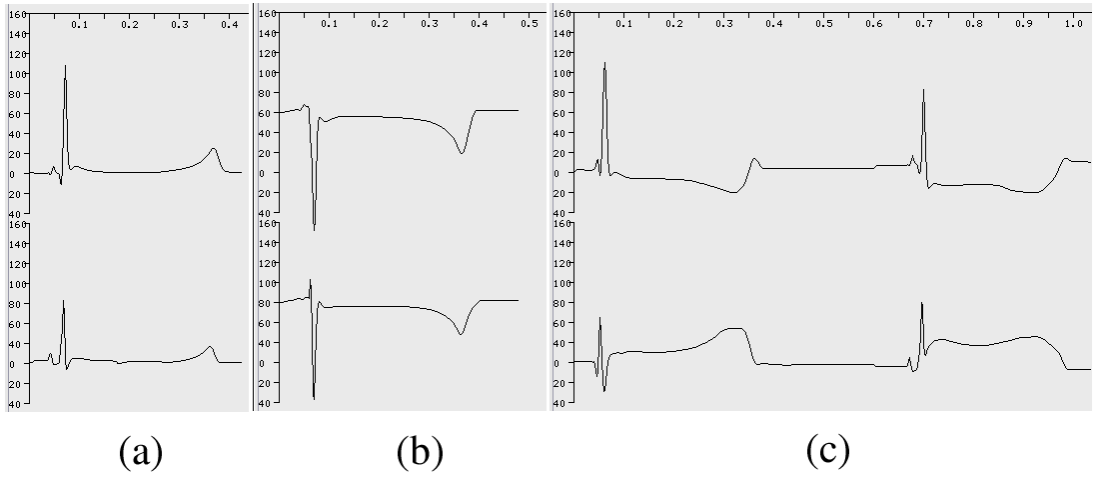
**The simulated ECGs with the 2-D model. **(a) The normal ECG; two leads are at the middle of left and right chests (<190,50 > and <-80,50 >). (b) The normal ECG; two leads are in the cardiac cavities (<75,50 > and <32,50 >). (c) The ECG of endocardial ischemia (top line) and epicardial ischemia (bottom line); two leads are at the same positions as in (A). Two ischemia areas are shown in Figure 6(c).

The data used to build the 3-D heart model are the axial images of the Visible Human Project (VHP) digital male cadaver [17], which contains about 125 thoracic slices at a 1 mm interval. Using an image processing program we developed, we enter a 128 × 128 coordinate system on each slice and use a computer mouse to mark the cells of different tissue with different colors (Figure 3). This process digitalizes each slice into a data file that reports the coordinates and type of each cell. The current heart model contains six kinds of cardiac tissues: sinoatrial node (SAN), atrioventricular node (AVN), atrium (AT), ventricle (VT), and trunk conduction bundle in the atrium (CBA) and in the ventricle (CBV). The distribution of trunk conduction bundles in each slice is determined by clinical experts, but the terminal distribution of the Purkinje fibers is generated by an algorithm at runtime. After processing all 125 slices, we use a program to merge the generated 2-D data files into a 3-D data file, which constitutes the anatomical model of the heart that occupies about 280,000 cells in the 128 × 128 × 128 cellular automata cell space. We also build an illustrative 2-D model containing the same kinds of cells and the same action potential models to evaluate the ECG simulation algorithms (Figure 6). The simulated normal and some abnormal ECG waveforms support the validity of the algorithm (Figure 7).

### Heterogeneity, anisotropy and inhomogeneity

The heterogeneity of a model is described in two ways. First, a specific field *type*, indicating cell type, is defined in the cell program, but whose value is initially stipulated in the anatomical model when we process tissue slices, such as:

[x, y, z] = type, ......

x, y, and z are the coordinates. Values of other fields can be defined or not in the data file. With a nested *if-then *statement on the value of *type, *the cell program is divided into several parts, each being executed by cells of the specific type. Second, *type *can be modified at runtime to simulate pathological changes. After a cell type is changed, its program and therefore its behavior also change.

In a tissue or organ, aside from heterogeneity, cells often show anisotropy and inhomogeneity that cannot be conveniently described while processing the raw data. To describe these two properties needs a bit of pattern formation programming [24], an interesting and challenging issue of biological modeling with cellular automata. In building the whole-heart model, the first relevant issue is cells at different layers in cardiac walls have different electrical properties [16,25]. To express this inhomogeneity, we developed a resolution-and dimension-independent algorithm to stratify cardiac walls into layers at the initial stage of runtime. The algorithm is comprised of three steps, and is run by all cells:

Let the layer number of SAN cells (at least one SAN cell is on the epicardium) be 0 and the layer number of AVN cells (at least one AVN cell is on the endocardium) be 50.

• If the current cell is neither a ventricular nor an atrial cell, then: if it connects to a cell whose layer is 0, its layer is 0; if it connects to a cell whose layer is 50, its layer is 50.

• If the current cell is a ventricular or an atrial cell, then: visit all its neighboring cells and find out their minimal layer number, *Min*. The layer of this cell is *Min+1*.

A slice of the stratified 3-D model and of the stratified 2-D model is shown in Figure 6. After stratification, the layer number of each ventricular cell is added to its HH equations as a special parameter to control and adjust its action potential duration (we do not use the layer number of atrial cells). This procedure ensures that epicardial cells have shorter action potential duration than endocardial cells, and the epicardium-to-endocardium repolarization is naturally and faithfully established. By assigning a large layer number to cells in the middle layers of the ventricular walls, an unusually long action potential duration is created, and the specific electrical property of M cells can be naturally simulated [25].

The slices of the digital cadaver do not provide any useful information on the distribution of terminal Purkinje fibers. Even if we know that most of them are located in the subendocardium, it is impractical to make a slice-by-slice manual description. Based on the stratified cardiac walls, this issue can be easily solved by using a lateral inhibition algorithm applied to cells on the ventricle subendocardium to generate a mesh-like Purkinje network. On completion of running the algorithm, some of the cell types are changed from ventricular cell to Purkinje cell.

The more difficult engineering issue is the fine structure of cardiac cells and conduction fibers. Simulation with the 2-D model demonstrates that, even if the HH action potential model of Purkinje cells produces a very fast upstroke, which means a quick transjunctional conduction, yet a one-cell by one-cell conduction can never give normal propagation profiles as observed in Durrer's experiment, due to a too long transjunctional delay [26]. Nevertheless, our simulation shows that the rapid conduction in conduction fibers can be implemented by *n*-by-*n *cell communication among automaton cells of conduction fibers without physically building the fiber structure; *n *= 4 gives very satisfactory simulation results. Again, we propose that by using a pattern formation algorithm the physical construction of fiber structures is also feasible. In this scenario, a fiber is assembled by *n *successive automaton cells sharing the same, unique identity number, among which there is no conduction delay. Currently, with the 2-D model we find that even if letting each automaton cell stand for a discrete ventricular cell, the model works quite well in ECG simulation.

### Computation within and between cells

**Figure 4:**
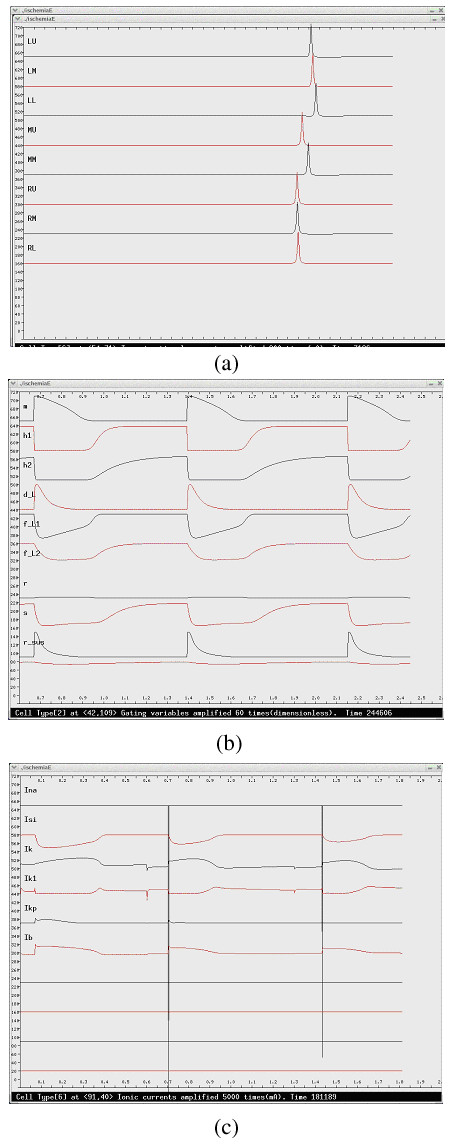
**The channel level electrical activities of a ventricular cell. **(a) The transjunctional currents from eight neighboring cells. (b) The state of gating variables. (c) The transmembrane ionic currents.

**Figure 5:**
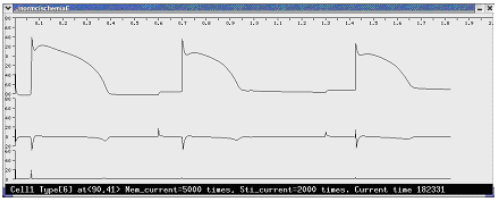
**The cell level electrical activities of a ventricular cell. **The top line is the transmembrane potential; the middle line is the transmembrane current; the bottom line is the stimulating current received from neighboring cells. The cell is under progressive ischemia, which can be reflected in the change of action potential.

Each automaton cell is a computing unit for action potential and ECG simulation. The electrical activity of each automaton cell of a specific cell type is described by the corresponding HH type action potential model. Five action potential models are employed to simulate activities of different cardiac cells (The cells of trunk conduction fiber in atriuma and ventricles use the action potential model of the Purkinje cell) [27-31]. Since the models are based on experimental data from different animal species, parameters of K^+ ^channels are slightly changed to produce action potential duration of human cardiac cells. Time constraints are the only reason for us to adopt early published, simpler action potential models. In the given simulations, all action potential models are solved using explicit Euler integration with a time step, Δt, of 0.01 ms [32]. An asynchronous adaptive time step method has also been developed to speed up simulation [49]. Electrical activities at channel (Figure 4), cell (Figure 5), and organ (Figures 6, and 7) levels are monitored and captured at runtime.

Each automaton cell in the 3-D model has 26 neighbors, linked by gap junctions. When a cell depolarizes, driven by the potential difference between it and its neighboring cells, transjunctional currents generate and propagate to neighbors through gap junctions. Simulations with the 2-D model show that the simple static gap junction model, in which the resistance of the gap junction is a constant and the transjunctional current follows Ohm's law, works quite well. The dynamic gap junction models, in which the resistance of gap junction changes in a nonlinear manner with membrane potential, will significantly increase computational time [33,34] and create an extra burden for large-scale modeling.

Values of gap junction resistance between different cells and in different directions are initially set according to available experimental data, and then tuned via simulation according to Durrer's experimental observations [26]. Excitation conduction between cells of the same layer follows an end-to-end propagation, and between cells of different layers is a side-to-side process. Parameters are also adjusted to fix the ratio of side-to-side conduction speed vs. end-to-end conduction speed to be one-third in ventricular cells and one-tenth in atrial cells [35]. Gap junction resistance can be modified at runtime to examine its effect on excitation propagation. For each cell in each round of computation, before solving the HH equations, the transmembrane potentials of all neighboring cells are checked and the transjunctional currents computed and summed to get the stimulating current, *I*_*stim*_, that the cell receives. The direction and speed of electrical propagation within the heart are jointly controlled by: (1) the stratification of the ventricular walls, (2) the speed of end-to-end and side-to-side conduction, (3) the ratio of end-to-end conduction speed to side-to-side conduction speed, and (4) the distribution of the trunk conduction bundle.

### Description of pathological activity

In the cardiac model built with the extended cellular automata, abnormal electrical activities are grouped into four classes. The first class is based on anatomical defects in the heart that can be described by changing the type of some cells, either in the anatomical model or in the cell program. The Wolff-Parkinson-White syndrome caused by atrium-ventricle bypasses is a representative case. The second class is resulted from aberrant environments, especially abnormal ionic concentration (e.g. hyperkalemia) which significantly influences action potential. Abnormality of transjunctional conduction constitutes the third class, and the fourth class is is comprised of anomalous dynamics of cellular electrical activity itself. Changes in the HH equations can simulate these aberrations. In most cases, pathological changes affect more than one aspect. For example, in addition to providing an abnormal cell environment, ischemia also results in changed cellular electrical activity and altered gap junction resistance. To simulate the effect of ischemia, a special field *blood, *with normal value 1.0 is defined in the cell program, and introduced as an extra parameter in action potential and in gap junction models. To multiply the conductivity of the gap junction by an abnormal value of *blood *(e.g., *blood* 0.7) can simulate a lowered gap junction conduction speed. A small value for *blood*, when inserted into the equation computing Ca^++ ^current, affects the generation of the action potential. Dynamically modified *blood *can naturally simulate progressive ischemia (Figure 5 and Figure 7) [36,37].

Combining these factors, a variety of arrhythmias can be simulated. For example, by partially or completely blocking the activity of cells, or the conduction of gap junctions at specific locations, various conduction blocks such as AV (atrium-ventricle) block, LBBB (left bundle branch block) and RBBB (right bundle branch block) can be conveniently simulated. Many rapid arrhythmias are triggered by successive ectopic beats. Such abnormal beats can be produced in the model by providing cells at specific locations with extra stimulation or by changing them from atrial/ventricular to SAN cells. For abnormal propagations such as long QT syndrome, caused by the extra long refractory period of M cells, we can adjust, either dynamically or statically, the layer number of cells at the middle layers of the ventricular walls. Complex spatiotemporal patterns of arrhythmias are formed through discrete cell-cell communication. By these means, arrhythmic electrical activities can be simulated in flexible ways, different from those models created by cable equations or other partial differential equations (PDE).

### ECG simulation by computing field potential of every cell

Linking ECG simulation directly with cellular and channel electrical activity is crucial for the understanding and treatment of arrhythmias. Since each automaton cell in our model is a computing unit, a cell level ECG simulation algorithm is developed to compute each cell's field point potential. The algorithm, implemented as a backend function coded in C language, shows strengths, because such cell level simulation builds a link between the action potentials of cells and the ECG waveforms. The limitation here is that this heart model does not include the chest, so that the contribution of thoracic tissues to body surface potential is much simplified. The model reads the membrane potential of every automaton cell and computes its field potentials at the standard lead locations.

Several assumptions are made in the computations on account of established physical laws. (a) Each (automaton) cell has a spherical shape. Thus, the transmembrane potential, *V*_*m*_, is uniform on all parts of the cell membrane, except at gap junctions. (b) Gap junctions occupy the same area and are symmetrically distributed on each cell. (c) The distance from cell to field point is long enough so that the two solid angles subtended by the positive and negative sides of a cell membrane to a field point are equal. (d) Dielectric effects on field potential are neglected. σ_*media *_is the average conductivity of the 1-D tissues between a source cell and a field point; σ_e _the conductivity of the intercellular matrix; and σ_*i *_the conductivity of the intracellular cytoplasm. (e) For any neighboring cells, *j *and *k*, σ_je _= σ_ke_, σ_ji _= σ_ki _and φ_je _= φ_ke_, φ_e _is the potential in intercellular space. For convenience, we present the algorithm for the 2-D model here; the 3-D case is similar. The equation computing the field potential of an isolated cell at field point P is [38]



σ_i _φ_i _- σ_e _φ_e _is the double layer strength of the cell membrane. When there is no propagation on the cell membrane, the field potential is zero. If the cell connects with eight adjacent cells, equation (1) becomes:



Here, σ_i _φ_i _- σ_ji _φ_ji _is the double layer strength of the gap junction between the cell and its *j*th neighbor. The transjunctional potential difference between the two cells then becomes:

φ_i _- φ_ji _= φ_i _- φ_e _+ φ_e _- φ_ji _= φ_i _- φ_e _+ φ_je _- φ_ji _= V_m _- V_jm _    (3)

**Figure 8:**
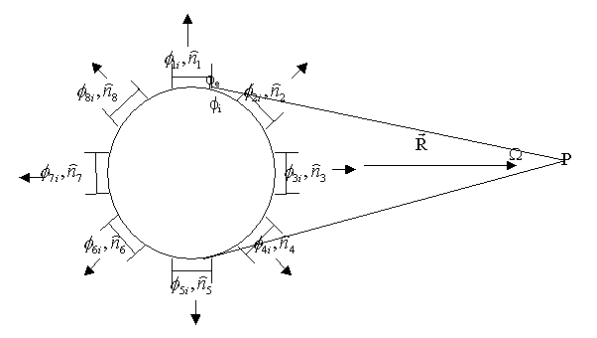
**Computing the field potential of single connected cells. **This figure shows the distribution of gap junctions on the membrane of cells in a 2-D cell array and how gap junctions contribute to field potential. φ_*i *_is the potential within the current cell; φ_1*i *_is the potential within the first neighboring cell;  is the normal direction of the transjunctional potential difference between the current cell and its first neighboring cell; ... Ω is the solid angle subtended by the current cell at field point P.



S1 to S8 are areas of the eight gap junctions; Ω_1 _to Ω_8 _are solid angles subtended by them; and S0 denotes the remaining part of the cell membrane (Figure 8). Let the membrane area positive to P be S_B _and the area negative to P be S_A_. If: (a) S_B _= S_A_, (b) the same and even number gap junctions distribute symmetrically on S_B _and S_A_, and (c) all gap junctions have an equal area, then it can be proved that:

Here, Σ K_A _is the sum of areas of gap junctions on S_A_, and Σ K_B _is the sum of areas on S_B_. In this circumstance, the first term in equation (2) is zero, and only the second term makes a contribution to field potential. The equation now becomes:



Considering all Sj (j = 1...8) to be equal, and cos α_1 _= cos α_5 _= cos 90° = 0, we have:



Here,  is a constant that affects only the baseline, but not the shape of the ECG waveform. The field potential at point P, generated by the whole heart, is the superposition of all cells' contributions:



Here, *n *is the number of automaton cells, and φ_j_(P) is computed with equation (5).

Due to the time needed for the numerical solution of massive HH equations, the computing of σ_media _R^2 ^has to be much simplified, as described in equation (7), where σ_media_i _is the conductivity of tissue *i*, and R_i _is the length of tissue *i *on the line between the source cell and the field point P.



As noted above, both the boundary effect and order of dielectric distribution are neglected. The 2-D (3-D) boundary between two tissues is treated as a 1-D boundary, and the ordered dielectric distribution is treated as an unordered distribution. Because of the huge number of cardiac cells and various possible field points on body surface, epicardium, endocardium and cardiac cavities, it is impossible to deal with numerous situations of boundary conditions between cells and field points. The method we adopt here is to make a runtime traverse from the source cell to the field point and meanwhile check the conductivity of each tissue and determine the 1-D space it occupies. The scaled distance is 1.0 between two perpendicular-connected cells, and 1.414 between two oblique-connected cells. Although the electrical property of non-cardiac tissues is much simplified, the electrical property of cardiac cells is intensively considered. Cardiac cells under different conditions have different conductivity [39]. It is σ_Depo _= 0.4 mhos/m in depolarization, σ_Repo _= 0.25 mhos/m in repolarization, σ_Rest _= 0.2 mhos/m in resting state, σ_Infarct _= 0.1 mhos/m in acute infarcted area, and σ_Ischemia _= 0.16 mhos/m in ischemic area. We let σ_Blood _= 0.6 and σ_Chest _= 0.1 be the average conductivity of tissues in chest [40,41]. We find ECG simulation is not clearly impaired by this simplified dielectric description, but benefits from the intensified cardiac tissue description.

## Results

### Factors that influence performance of the parallelized cellular automata model

For the physically parallelized whole-heart electrophysiological model built with the extended cellular automata, several factors impair the simulation efficiency on a cluster of SMP.

The first difficulty is the graphic display of the simulation. The main display window, as the critical resource in the parallelized program, can only be sequentially accessed by the cells. Thus, to display the updated state of all cells leads to a significant decrease of running speed. We mitigate this problem by displaying the cell state every 20 or even 50 steps, instead of every single step. The second problem is the overhead of communication among *slaves *located on different nodes. The time cost of communication, spent on the exchange of boundary sheets between neighboring *slaves *in each round, rises with the increase of *slave *number. An extreme case for a 128 × 128 × 128 model is that there are 128 nodes, and each *slave *deals with just one sheet, requiring every sheet to be exchanged in each round. The physical link between nodes also significantly affects performance. An additional limitation is that the overhead of *fork/join *operations in the OpenMP parallelized program degrades performance, although not very significantly. The OpenMP directive *parallel *can be inserted either before the endless *while *loop, or more simply, before the three *for *loops. If it is inserted before the *for *loops, the *fork *and *join *operations, which create and delete threads and allocate and collect memory for temporary variables in each thread, will be repeatedly executed in each round. The preferable way, which eliminates this unnecessary expenditure, is to insert the directive *omp parallel *before the *while *loop, and the directive *omp for *before the *for *loops. Between the *omp parallel *and the *omp for*,*omp master *is used to limit parallelism to only the cell program part. Finally, load balance becomes a limitation when a model has an irregular and/or heterogeneous structure. The heart, with four chambers and an uneven shape, is a typical case. Usually, on a cluster consisting of *m *identical nodes each containing *n *CPUs, a program is evenly dispatched to distribute the cell space across all nodes. We find that using this strategy, the 3-D heart model cannot reach the best load balance and performance because different nodes deal with different numbers of cardiac cells. Furthermore, even if each node contains the same number of cardiac cells, since different cells run different action potential models, the burden of computation remains unequal. Only a solid cubic model with homogeneous cells occupying the full cell space can ensure a best performance. Thus, if a model needs to run many simulations, an important issue is to find the best cell space partition.

### Evaluation of the 3-D cardiac model

**Figure 9:**
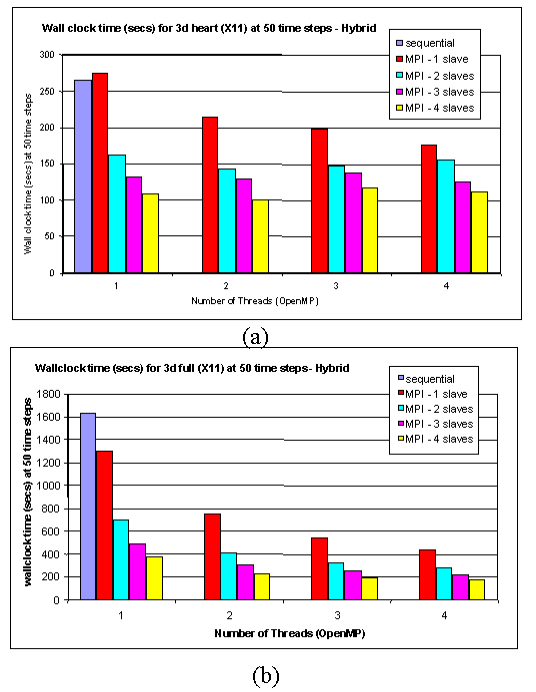
**The performance of running the 3-D model on a cluster of SMP nodes. **(a) When the 3-D cell space is sparsely occupied by the heart model (left), the evenly dispatch strategy does not produce the best performance. (b) When the cell space is fully occupied by ventricular cells (right), the best performance is guaranteed.

Pilot runs with the 3-D model have been made on a 4-node SUN computer cluster. The head node has eight UltraSparc CPUs, and the other nodes each have four identical CPUs. Five processes, 1 *master *and 4 *slaves*, are created at each run. The *master *and a *slave *are assigned to the head node, and each remaining *slave *is assigned to a node. Within each *slave*, four threads are created using the OpenMP *parallel *directive. Figure 9 gives the performance results for an even partition strategy. With this partition, due to the irregular structure of the heart and the heterogeneity of cardiac cells, the combination of 4 *slaves*-4 threads does not give the best performance. The 4 *slaves-*2 threads setting behaves better because the overall cost is lower. . However, when the whole cell space is occupied by ventricular cells, the 4 *slaves*-4 threads version provides the best performance, as predicted.

## Discussion

Arrhythmias are a group of complex syndromes not well understood. Many difficult issues exist when investigating arrhythmias through computational modeling. We cover here only a few relating to model building and running.

We adopt early published action potential models in the current 3-D model because they are simpler and more computationally affordable. In the first version of the Luo-Rudy model [30], there are six ionic channels, and the fluctuation of ionic concentration is not described. In the second version, there are many more channels, and the dynamic ion concentrations are described by another large group of ODEs [42]. It is straightforward to upgrade the cardiac model by replacing old action potential modelswith new ones. However, action potential models are not the whole story for a cardiac model. One has to make a compromise between the complexity of action potential models and the resolution of the cardiac model, because the computational burden also is due to the latter. Pollard *et al*. [43] in 1993 reported a cardiac model containing 400,000 computing nodes, but a much simplified action potential model was used [44]. This strategy was also adopted by other modelers [45]. We argue that full action potential models are important for modeling and understanding arrhythmias, especially those triggered by abnormal ionic electrical activities. However, to simulate more complex spatio-temporal cardioelectrical activities such as spiral waves in fibrillation, the current resolution of the model is undoubtedly insufficient. Technically, it is not difficult to adopt a higher resolution with more precise data, such as the digital female cadaver slices, which have an interval of 0.33 mm [17]. To double the resolution using a 256 × 256 × 256 coordinate system is another choice. By either method, the cell program, the pattern formation algorithms, and the ECG simulation algorithm remain the same – they are resolution-independent. This is a prominent and beneficial feature of such cellular automata modeling.

Although experimental evidence show that fiber orientation takes a role in arrhythmia generation [46,47], to implement a highly realistic description of fiber orientation is expensive because of the difficulty in acquiring sufficient validated data [48]. Encoding these data into models is also quite complex, requiring the use of complex mathematical tools such as tensors. In our model, a new approach is proposed for stratifying heart walls. If fibers are simply built with cells of the same layer, they automatically acquire an arc shape (Figure 6d) that can assume various orientations. Comparison of effectiveness between the two methods is currently not available due to the lack of sufficient simulations.

Another issue relates to ECG simulation. Although simulation results of the 3-D model have not been acquired thus far, the simulated ECGs with the 2-D model in different lead locations and under physiological and pathological conditions are impressive and qualitatively agree with recorded ECGs, supporting the validity of the algorithm. Theoretically, the boundary effect of dielectrics is not negligible for field potential computing; yet in practice simplifications are often inevitable. So far we find that our simplified treatment of boundary effect on field potential does not visibly affect ECG simulation. There may be two reasons for this. First, the conductivity of the tissues (not including the heart) in the chest may not be significantly different. Second, the boundaries among different tissues are so irregular that the net boundary effect may be effectively neutralized. On the other hand, we find that to let cardiac cells under different conditions (depolarization, repolarization, resting state, and ischemia) have different conductivity can improve the ECG quality, indicating that the precise description of the source may be more important than the precise description of the dielectric.

Finally, we point out that, as in single cell models where adaptive time steps can greatly improve the running performance, this same strategy can produce the same results in multicellular models built with this cellular automaton [49]. Simulation with the 2-D model shows that an overall speed improvement of 4.5 is reached.

## Conclusions

Effective simulation of arrhythmias needs a whole-heart model, differential description of electrical properties of cardiac cells, membrane equation based computation, association between cellular activities and ECG generation, flexible description of pathological conditions, and long running time. To comprehensively address these issues, we develop a method based on cellular automata and parallel computing technologies to build large-scale electrophysiological models with extended cellular automata, and run such models on clusters of shared memory machines. The dynamically traced and captured electrical activities at channel, cell, and organ levels can substantially help us understand abnormal cardioelectrical activities through simulation. Simulation results with the 2-D model support the validity of the ECG simulation algorithm. Transparent cluster computing is a convenient and effective solution to the excessive time consumption of computational intensive simulation.

In addition to reaching a mechanistic understanding of arrhythmias, an important goal of *in silico *research is to facilitate the discovery and evaluation of drugs. This helps to reduce the risk and cost of clinical trials, shorten the cycle of development, and remove randomness in candidate screening. A whole-heart electrophysiological model that links electrical activities at channel, cell, and organ levels can help achieve this result. The modeling method described in this paper shows the advantages of precisely linking cell and organ activities, exploiting the intrinsic parallelism in tissue/organ level biological activities. Besides modeling electrical activity, the method is also applicable to many other multicellular models in which quantitative description is required.

## Authors' contributions

HZ develops the methods and built the initial 3-D model. AM provides data of conduction system distribution in ventricles. YS and GR help solve some technical issues. PD is responsible for the project.
